# One-year outcomes of 27-gauge versus 25-gauge pars plana vitrectomy for uncomplicated rhegmatogenous retinal detachment repair

**DOI:** 10.1186/s40942-019-0164-0

**Published:** 2019-06-04

**Authors:** Giancarlo Sborgia, Alfredo Niro, Luigi Sborgia, Maria Oliva Grassi, Samuele Gigliola, Mario R. Romano, Francesco Boscia, Alessandra Sborgia, Giovanni Alessio

**Affiliations:** 1 Department of Medical Science, Neuroscience and Sense Organs, Eye Clinic, University of Bari, Bari, Italy; 2 Eye Clinic, Hospital “S. G. MOSCATI”, ASL TA, Via per Martina Franca, 74010 Statte, Taranto, Italy; 3 Department of Ophthalmology, Humanitas University, Pieve Emanuele, Milan, Italy; 4 Department of Surgical, Microsurgical and Medical Sciences, Eye Clinic, University of Sassari, Sassari, Italy

**Keywords:** 27-Gauge, 25-Gauge, Transconjunctival sutureless vitrectomy, Rhegmatogenous retinal detachment, Surgical time, Vitrectomy time

## Abstract

**Background:**

27-gauge (27G) and 25-gauge (25G) transconjunctival sutureless vitrectomy (TSV) were considered equal about safety, effectiveness and vitrectomy time for the treatment of rhegmatogenous retinal detachment (RRD), although larger and long-term comparative studies are needed to confirm previous knowledge. Furthermore, a combined comparison of time duration of surgery and vitreous removal was never performed. Our purpose was to compare the safety and efficacy of 27G versus 25G TSV for the treatment of uncomplicated RRD over a 1-year follow-up.

**Methods:**

A 12-months single-center prospective, randomized, interventional study of 92 consecutive patients was performed. 46 patients underwent 27G TSV (Group 1) and 46 underwent 25G TSV (Group 2). Primary outcomes were primary and final reattachment rate, and final functional success (visual acuity ≥ 20/200, 1 LogMar). Secondary outcomes were the surgical and vitrectomy time. Complications were recorded.

**Results:**

All functional and morphologic data at baseline and at all follow-up time points up to 12 months after surgery were available for only 88 patients. Four patients in Group 1 dropped out of the study after surgery. There was no significant difference in baseline characteristics between the two groups. Primary and final reattachment rates were 90.5% and 100% in Group 1, and 95.6% and 100% in Group 2, respectively (*p* > .05, *p* > .05, respectively). Visual acuity improved from 1.5 ± 1.09 LogMar to 0.38 ± 0.55 LogMar in Group 1 (*p* < .001) and 1.2 ± 0.9 LogMar to 0.49 ± 0.53 LogMar in Group 2 (*p* < .001), without significant difference between the groups (*p* > .05). The surgical time was 73.2 ± 11.3 min with 27G TSV and 64.4 ± 9.5 min with 25G TSV (*p* = .0001). The vitrectomy time was 19.9 ± 3.8 min with 27G TSV and 20.8 ± 3.8 min with 25G TSV (*p* > .05). One single case of choroidal detachment occurred.

**Conclusions:**

Reattachment rates, functional success and vitrectomy time were comparable between 27G and 25G TSV for RRD. Surgical time was significantly longer using 27G vitrectomy.

## Background

Since the introduction of pars plana vitrectomy in early 1970 by Machamer et al. [1], as alternative to the scleral buckling surgery to treat rhegmatogenous retinal detachment (RRD), the innovation trend has been moving in the direction of smaller instrument calibers. About this surgical technique, Chen et al. [2] proposed a sutureless approach to decrease surgical trauma and postoperative inflammation. Fujii et al. [3, 4] introduced the 25-gauge transconjunctival sutureless vitrectomy (TSV). Three years later, Eckardt [5] developed the 23-gauge TSV in order to reach a compromise between the more traumatic 20-gauge and too flexible 25-gauge. Recently, innovations like more powerful light sources, rigid materials for small gauge instruments and more efficient vitrectomy machines with better fluidics and controls, allowed the progression to the 27-gauge (27G) vitrectomy system [6–8].

The progressive reduction of the size of the probes associated with high-performance vitrectomy allowed better postoperative comfort and earlier visual recovery for patients affected by RRD.

Oshima et al. [9] first reported a preliminary study regarding the safety and practicality of the 27G system for the vitreo-retinal surgery. Several reports have suggested the clinical outcomes and short-term safety profile of 27G TSV for many vitreo-retinal surgical indications, including RRD [10–13].

In two prospective 6-months follow-up studies, Romano et al. [14] and Rizzo et al. [15], comparing 27G and 25G TSV, reported that these two systems were equal about safety, effectiveness and vitrectomy time for treatment of RRD but a combined comparison of time duration of surgery and vitreous removal was not performed.

Furthermore, larger and long-term comparative studies would be needed to confirm suggested data.

We conducted a 12-months prospective randomized interventional study to compare clinical outcomes, surgical and vitrectomy time, and complications between 27G and 25G TSV to repair RRD.

## Methods

A single-center prospective randomized comparative study was performed on 92 consecutive patients with RRD between April 2016 and June 2017.

Patients who met all of the following criteria were considered for inclusion in the study: RRD with one or more retinal breaks and the ability to give informed consent. Patients who met any of the following criteria were excluded from study entry: a history of any previous vitreoretinal surgical procedures or penetrating ocular trauma, proliferative vitreoretinopathy (PVR) of grade C or higher, significant ocular comorbidities such as uveitis, uncontrolled glaucoma, amblyopia, severe non proliferative or proliferative diabetic retinopathy, and inability to maintain postoperative posturing. All patients underwent preoperative evaluation included complete medical, surgical, and ophthalmic history. Preoperative data included age, sex, axis length, lens status, macular status, extent of retinal detachment, number of breaks, best-corrected visual acuity (BCVA), days from visual loss to surgery and intraocular pressure (IOP). Patients were divided in two groups. In Group 1, 46 patients were treated with 27G TSV and in Group 2, 46 patients underwent 25G TSV. All surgeries were carried out by one surgeon G.S. in Eye Clinic, Department of Ophthalmology, University of Bari, Italy.

A postoperative examination was carried out on 2 weeks and 1, 3, 6 and 12 months after surgery, and included visual acuity measurement (BCVA), biomicroscopy, intraocular pressure (IOP) measurement, funduscopy and optical coherence tomography (OCT) analysis. IOP measurement was also performed at 1 day after surgery.

Main outcomes were: the primary anatomical success as a complete reattachment of the retina following a single procedure when all the gas in the eye had disappeared; the final anatomical success including the primary anatomical success as well as those patients for whom a reattached retina was achieved with subsequent surgery over 1 year follow-up; the functional success considering BCVA ≥ 20/200 (1 LogMar).

Secondary outcomes were the surgical time (the time period between trocars positioning and control of sclerotomies after trocars removing) and the vitrectomy time (the time period when the cutter was activated for removing the vitreous) recorded by the vitrectomy machine. At the end of surgery intraoperative complications were recorded.

Visual acuity was recorded after gas bubble got smaller not limiting central vision. BCVA was recorded as a Snellen visual acuity and converted to logarithm of minimal angle of resolution (logMar) units for statistical analysis. Counting finger (CF) vision was defined as 2.0 logMar and hand movements (HM) were defined as 3.0 logMar. OCT scans using 6 × 6 radial scans protocol by OCT AVANTI RTVUE XR (OPTOVUE, Fremont, CA, USA) were recorded starting from the third month. IOP was measured using Goldmann applanation tonometry, and severe postoperative ocular hypotony and hypertony were defined as IOP < 6 mmHg and IOP > 30 mmHg, respectively.

After each patient signed the informed consent form, their identification number was recorded and patients were randomly assigned into Group 1 or Group 2 using a computer-generated randomization list. A total of 92 patients underwent surgery and met inclusion criteria. All functional and morphologic data at baseline and at all post-baseline time points up to 12 months after surgery were available for only 88 patients. 4 patients in Group 1 dropped out of the study after surgery.

The study followed the tenets of the Declaration of Helsinki and was approved by the institution’s review board.

### Surgical technique

Before starting surgery, the eyelid and periorbital skin were prepared with 5% povidone-iodine (Betadine; Purdue Fredrick Co, Norwalk, CT). All surgeries were performed under a retrobulbar block (a mixture of 2% Lidocaina and 2% Mepivacaina), using the Constellation^®^ vitrectomy system (Alcon, Fort Worth, TX, USA). For this study, the machine was set with an initial aspiration of 0 mmHg moving linearly to 650 mmHg when the foot pedal is fully depressed, maintaining a fixed cut rate of 7500 cuts per minute (cpm) in both vitrectomy systems (27+ and 25+ Total Plus Pak). During surgery, IOP was controlled to 25 mmHg. For posterior visualization, surgical microscope Resight 700 (Carl Zeiss Meditec AG, Oberkochen, Germany) was used. Both the 27G and 25G procedures were performed using a three-port transconjunctival pars plana approach.

After the conjunctiva was displaced slightly, the trocars were placed through the conjunctiva and the sclera 3.5 mm from the limbus using a 30 degree angled biplanar approach to create a self-sealing incision (as suggested by Shimada) [16]. Phacoemulsification was performed in all phakic eyes to maximize the complete removal of the anteroperipheral vitreous. After core vitrectomy, if needed, posterior vitreous detachment was induced and complete removal of the vitreous gel was performed. Triamcinolone acetonide was routinely injected to facilitate visualization of the vitreous base which was meticulously shaved circumferentially with scleral indentation. Endolaser photocoagulation was performed to treat any detected tears or suspicious retinal lesions.

Transvitreal diathermy was used, at need, to mark retinal breaks and achieve intraocular hemostasis. If needed, intraoperative use of perfluorocarbon liquids (PFCL) was chosen during the procedure. After air-fluid exchange, 22% sulfur hexafluoride (SF6) gas or 14% perfluoropropane (C3F8) gas were used as tamponade. At the end of the surgery, the microcannulas were removed and a gentle massage of the sclerotomy with a cotton-tipped applicator was applied to facilitate self-sealing and avoid leakage. If persisting leakage was detected, 8-0 vicryl sutures were placed in the wound and the overlying conjunctiva. Either 3 days face down positioning or one side pose depending break positioning were assigned to the patient. All patients were placed on antibiotic and betamethasone eye drops therapy with tapered frequency during the 5 weeks after surgery. During the follow-up period if IOP was higher than 25 mmHg, antiglaucoma eye drops were prescribed.

### Statistical analysis

The qualitative variables are presented as frequencies and percentages, while quantitative data as means ± standard deviations. Effectiveness of the treatment was assessed with a *t* test on morpho-functional changes (paired 12 months post-surgery value minus pre-surgery value).

Differences between the groups were assessed using Pearson’s Chi squared for categorical variables and Kruskal–Wallis test or One-way Analysis of Variance (ANOVA) followed by post hoc Tukey’s HSD test for quantitative ones.

The sample-size was determined considering a confidence level of 95% and a confidence interval of 10%. *p* value < 0.05 was considered statistically significant. All statistical analyses were conducted using R (v 3.3.1) and Rstudio (v 1.0.153).

## Results

### Preoperative Characteristics

Table 1 summarizes patients’ demographic and baseline clinical data. Forty-two eyes underwent 27G TSV (Group 1) and forty-six eyes underwent 25G TSV (Group 2) were considered in the analysis. The age of the patients ranged from 39 to 75 years in the Group 1 and 38 to 78 years in the Group 2. There were 31 men and 11 women in the Group 1 and 35 men and 11 women in the Group 2 (*p* = 1). Mean time from visual loss to surgery was 8.5 ± 4.4 days (range: 2–21 days) in the Group 1 and 7.9 ± 4.9 days (range: 3–22 days) in the Group 2 (*p* = .56). There was no statistical difference in lens status between both groups (*p* = .89). A posterior vitreous detachment (PVD) was detected intraoperatively in 31 eyes and 32 eyes in Group 1 and 2, respectively (*p* = .83). The mean number ± SD of retinal breaks was 1.8 ± 0.9 (range: 1–5) in Group 1 and 1.5 ± 0.7 (range: 1–4) in Group 2 (*p* = .07). Thirty-two eyes had RRD involving one or two quadrants and ten eyes had more extensive RRD involving three or four quadrants in Group 1. Forty-two eyes had RRD involving one or two quadrants and four eyes had more extensive RRD involving three or four quadrants in Group 2 (*p* = .22). The macula was attached preoperatively in 42.8% of eyes in Group 1 and 47.8% in Group 2 (*p* = .8). PVR grade ≤ B was preoperatively reported in 10 (24%) eyes in Group 1 and 14 (30%) eyes in Group 2 (*p* = .6). Preoperative mean IOP was 14 ± 2.1 mmHg and 15 ± 2.9 mmHg in Group 1 and 2, respectively (*p* = .89).

**Table 1:** Demographic and baseline clinical data

	Group 1 (27G TSV) n° 42	Group 2 (25G TSV) n° 46	p*
Sex (man/woman), number (%)	31/11 (74/26)	35/11 (76/24)	1
Age (years ± SD)	59.9 ± 9.2	61.7 ± 8.7	.35
Axial length (mm ± SD)	24.38 ± 1.68	24.46 ± 1.57	.82
Lens status (Phakic/pseudophakic), number (%)	24/18 (57/43)	28/18 (61/39)	.89
Quadrants of retinal detachment, 1/2/3/4	20/12/5/5	30/13/2/1	.22
Number of retinal breaks (mean ± SD)	1.8 ± 0.9	1.5 ± 0.7	.07
Macula On/Off, number (%)	18/24 (43/57)	22/24 (48/52)	.8
PVR, number (%)	10 (24%)	14 (30%)	.6
PVD, number (%)	31 (74%)	32 (69%)	.83
IOP (mmHg ± SD)	14 ± 2.1	15 ± 2.9	.89

*PVR* proliferative vitreoretinopathy of grade ≤ B, *PVD* posterior vitreous detachment, *IOP* intraoculare pressure

p* values, comparison between Group 1 and Group 2; *p* values < 0.05 were considered significantly

### Main outcomes

#### Anatomical Results

The primary and final anatomical success rates were 90.85% and 100% in Group 1 and 95.7% and 100% in Group 2, respectively (*p* = .59, *p* = 1, respectively). In the 27G TSV group, one eye developed a retinal redetachment within 1 month, one eye within 3 months and 2 eyes within 6 months after first surgery. In the 25G TSV group, two cases detached within 3 and 6 months after primary vitrectomy, respectively. The redetachments were due to PVR in 2 eyes (1 eye in the Group 1 and 1 in the Group 2), a new retinal break in 3 eyes (2 eyes in the Group 1 and 1 in the Group 2), and the opening of an original retinal break in one eye in the Group 1. All these eyes were retreated using 25G TSV approach. Silicone oil tamponade was used in case of redetachment due to PVR, in the other cases gas tamponade was used. Silicone oil was removed after an average of 3 months after second surgery.

#### Visual acuity

Table 2 summarizes BCVA at each examination. Preoperative BCVA was 20/630 (1.5 ± 1.09 LogMar) in Group 1 and 20/320 (1.2 ± 0.9 LogMar) in Group 2 (*p* = .15). Six eyes underwent reintervention for redetachment, because of the use of 25G TSV in these cases, the silicone oil tamponade in some cases, and in general, the postsurgical condition, were not considered in measuring of visual recovery. Therefore, the change of postoperative visual acuity was compared between the two groups, considering 38 and 42 patients with primary anatomical success, respectively. Their postoperative BCVA at the last visit improved to 20/48 (0.38 ± 0.56 LogMar) in Group 1 (*p* < .001) and 20/62 (0.49 ± 0.53 LogMar) in Group 2 (*p* < .001), without significant difference between the groups (*p* = .38).

**Table 2:** Best corrected visual acuity over 12 months follow-up

	Group 1 (27G TSV)	Group 2 (25G TSV)	*p* *
Baseline BCVA *(logMAR) (mean* *±* *SD)*	1.5 ± 1.09	1.2 ± 0.9	.15
Postoperative BCVA^a^ (1 month)	0.54 ± 0.59	0.75 ± 0.50	.08
p** < .001			
Postoperative BCVA^a^ (3 months)	0.45 ± 0.68	0.51 ± 0.54	.68
p** < .001			
Postoperative BCVA^a^ (6 months)	0.43 ± 0.56	0.49 ± 0.54	.50
p** < .001			
Postoperative BCVA^a^ (12 months)	0.38 ± 0.56	0.49 ± 0.53	.38
p** < .001			

*p* values < 0.05 were considered statistically significant

*BCVA* best corrected visual acuity

p* values, comparison between Group 1 and Group 2

p** values, comparison between Baseline BCVA and Postoperative BCVA within single groups

^a^patients with primary anatomical success (38 eyes, Group 1; 42 eyes, Group 2)

### Secondary outcomes

#### Surgical and vitrectomy time

In order to maximize vitreous removal, phacoemulsification was performed in every phakic eyes. The mean duration of vitrectomy was 19.9 ± 3.8 min with 27G TSV and 20.8 ± 3.8 min with 25G TSV (*p* = .27). The mean duration of surgery was 73.2 ± 11.3 min with 27G TSV and 64.4 ± 9.5 min with 25G TSV (*p* = .0001). When PVD was absent, surgical time was 77.2 ± 12.1 min and 62.1 ± 6.6 min in Group 1 and 2, respectively (*p* = .002). When PVD was already present, surgical time was 71.8 ± 10.8 min and 65.4 ± 10.5 min in Group 1 and 2, respectively (*p* = .02).

PFCL was given in 12 eyes (28.6%) in Group 1 and 12 eyes (26.1%) in Group 2

In eyes filled with PFCL surgical time was 81 ± 9.77 min and 74.2 ± 8.9 min in 27G and 25G TSV, respectively (*p* = .08).

In both groups, all eyes received endolaser and no external cryoapplication was given. In Group 1, 27 eyes (64.3%) had SF6 gas tamponade and 15 eyes (35.7%) had C3F8 gas tamponade. In Group 2, 25 eyes (54.3%) had SF6 gas tamponade and 21 eyes (45.6%) had C3F8 gas tamponade.

After removal of the microcannulas, only one sclerotomy site in 2 eyes in the Group1 and in 3 eyes in the Group 2 was sutured because of leakage.

### Complications

No complications occurred related to phacoemulsification such as posterior capsule rupture or zonular dialysis.

In the 27G TSV group, we also experienced one single case of choroidal detachment by infusion cannula slippage into the suprachoroidal space because of a sudden movement of the patient during scleral depression. At 1 day the mean IOP was 14.5 ± 3.7 mmHg (range 8–16 mmHg) in Group 1 and 15.5 ± 2.8 mmHg (range 10–20 mmHg) in Group 2 (*p* = .14).

No iatrogenic retinal breaks (IRBs), such as severe hypertension (IOP > 30 mmHg) or hypotony (IOP < 6 mmHg), intraocular bleeding, or endophthalmitis, were noted in the follow-up period in either group. Over follow-up OCT scans revealed epiretinal membrane (ERM) in 5 patients (12%) in Group 1 and 2 patients (4.3%) in Group 2 (*p* = .5), and cystoid macular edema (CME) in 2 patients (4.7%) in Group 1 and 1 patient (2.1%) in Group 2 (*p* = .07).

## Discussion

Compared with the 25-Gauge or 23-Gauge vitrectomy systems, the 27-Gauge system inducing minimal ocular trauma with a smaller incision, may decrease the inflammatory response, and may allow for overall patient with RRD a faster recovery.

The main limits about 27-Gauge probes are related to the stiffness of the instruments, the capability to remove vitreous as much as possible, and the time to perform surgery and, in particular, a complete vitrectomy approaching a RRD. We found that the 27G TSV provided anatomical and functional results comparable to those obtained using the 25G TSV, as previous papers reported [14, 15, 17].

The primary reattachment rate was higher in Group 2 but in Group 1 a higher number of eyes (ten eyes in Group 1 vs. three eyes in Group 2) had more extensive detachments (more than 2 quadrants) at baseline. Anyway, the primary anatomical success rate did not differ significantly between the groups and was similar to those reported by previous papers ranging from 90% to 100% using 27G [10–15, 17] and 93% to 100% using 25G TSV [14, 15, 17–23]. In both groups retinal redetachment occurred within 6 months after first surgery.

As previous papers reported [14, 15, 17], in this study different small gauge approaches led to significant and similar visual acuity recovery over 12-months follow-up, probably also due to the short mean time of visual loss before surgery was performed and relatively high number of eyes with macula on RRD in each group (Group 1, 43%; Group 2, 48%). Our results showed that 27G TSV was as effective as 25G TSV in reattaching the retina after initial surgery and improving visual acuity.

As previous reports suggested [14, 15, 17], we found that the duration of vitreous removal was not different between the two different gauge systems.

During the vitrectomy, the aspiration efficiency of 27G TSV was obviously inferior to that of 25G TSV [23], but the increasing cut rate is related to a high vitreous flow rate [24]. At the same time, lower aspiration efficiency of the 27G TSV compared with the 25G TSV ensures a rather safe vitreous gel shaving and a better fluidic stability. The 27-gauge cutter takes a faster and smaller ‘‘vitreous bite’’ due to the high vacuum settings, allowing an adequate flow rate during the vitreous removal [25].

On the other hand, our results did show a significantly longer surgical time in 27G group (73.2 ± 11.3 min) compared to 25G TSV (64.4 ± 9.5 min). Mitsui et al. reported that in 27G TSV, the operation time was significantly longer than that in 25G TSV for the epiretinal membrane [26]. Romano et al. [14] and Otsuka et al. [17] did not found significant difference in operative time considering 27 or 25-gauge to treat RRD, but authors did not define exactly the time period considered as operative time. Furthermore, in the prospective study of Romano et al. [14], silicone oil implant was used in some eyes differently from our study.

As we found, different maneuvers as the induction of PVD, more frequently in younger patients, and the infusion and removal of PFCL may extend the operation time when we use 27-gauge instruments.

In our series five of seven eyes which developed postoperative ERM after one single successful surgery, underwent 27G TSV and did not have a preoperative PVD. Our results suggest that the development of a postoperative epiretinal macular membrane could be caused by factors related to smaller gauge instrumentations. Inducing PVD is more difficult with 27G instrumentation due to lower flow/suction, especially in younger patients whose posterior vitreous cortex is tightly adherent to the retina. Less aspiration may reduce the efficacy of vitreous delamination when PVD was induced, with persistence of pro-inflammatory factors staining in vitreous chamber or retinal pigment epithelial cells that escaped through retinal tears [27].

We found no significant difference in the number of wounds that required sutures between the two systems. Generally, 27G TSV requires a smaller incision, which suggests the possibility of excellent self-closing of the wound, compared to other vitrectomy systems with larger-gauges [9, 23].

We monitored IOP from 1 day to 12 months after surgery and did not found significant difference. The surgery was completed by filling the vitreous cavity with gas tamponade in all the cases. Previous reports have indicated that when a gas tamponade was performed, the postoperative IOP was more stable than when it was not used [9, 28].

In this case series, no serious intraoperative complications, such as iatrogenic retinal breaks/tears, posterior capsule touch, leakage of sclerotomies, were observed. Safety-related outcomes were comparable to or better than some previous reports [14, 15, 17–22].

Limitations to our study are the small number of the patients and the lens status of all patients before vitrectomy. Since all phakic patients were rendered pseudophakic before vitrectomy, the efficacy and safety of different gauges in phakic eyes would not be seen in our study, On the other hand, as points of strength of our prospective study we highlight that all the surgical procedures were performed by a single experienced surgeon by using a well standardized technique, the long-term follow-up, and the concomitant analysis of surgical and vitrectomy time.

## Conclusions

We performed a comparative study of outcomes between 27G and 25G TSV for RRD. The surgical results of the two groups were equivalent with a longer surgical time using 27-gauge cutter. We believe that 27G TSV is as useful as 25G TSV for uncomplicated RRD. A randomized, controlled trial with a larger number of patients is needed to confirm the results obtained in this study.
